# Gas Sensing Properties of Perovskite Decorated Graphene at Room Temperature

**DOI:** 10.3390/s19204563

**Published:** 2019-10-20

**Authors:** Juan Casanova-Cháfer, Rocío García-Aboal, Pedro Atienzar, Eduard Llobet

**Affiliations:** 1 MINOS-EMaS, Universitat Rovira i Virgili, 43007 Tarragona, Spain; juan.casanova@urv.cat; 2 Instituto de Tecnología Química, CSIC-UPV, Universitat Politècnica de València, 46022 Valencia, Spain; rogarab@itq.upv.es

**Keywords:** lead halide perovskite, graphene, gas sensing, NO_2_ detection, NH_3_ detection, room temperature sensor

## Abstract

This paper explores the gas sensing properties of graphene nanolayers decorated with lead halide perovskite (CH_3_NH_3_PbBr_3_) nanocrystals to detect toxic gases such as ammonia (NH_3_) and nitrogen dioxide (NO_2_). A chemical-sensitive semiconductor film based on graphene has been achieved, being decorated with CH_3_NH_3_PbBr_3_ perovskite (MAPbBr_3_) nanocrystals (NCs) synthesized, and characterized by several techniques, such as field emission scanning electron microscopy, transmission electron microscopy and X-ray photoelectron spectroscopy. Reversible responses were obtained towards NO_2_ and NH_3_ at room temperature, demonstrating an enhanced sensitivity when the graphene is decorated by MAPbBr_3_ NCs. Furthermore, the effect of ambient moisture was extensively studied, showing that the use of perovskite NCs in gas sensors can become a promising alternative to other gas sensitive materials, due to the protective character of graphene, resulting from its high hydrophobicity. Besides, a gas sensing mechanism is proposed to understand the effects of MAPbBr_3_ sensing properties.

## 1. Introduction

Atmospheric contamination is one of the most important environmental issues in current societies. According to the World Health Organization (WHO), air pollution is linked to 7 million premature deaths in 2012 [1]. The most common anthropogenic pollution sources are coming from industrial processes, automotive and energy production [2]. The gases emitted can produce an important greenhouse effect such as carbon dioxide (CO_2_), methane (CH_4_), and ozone (O_3_), or can be dangerous for human health above very low levels of exposure. Nitrogen dioxide (NO_2_) and ammonia (NH_3_) are two examples of these harmful species. For these reasons, the in-field monitoring of harmful gases is a mandatory goal to control dangerous levels of contaminants. Above the threshold limits, when human health is at risk, gas sensors can be the last wall to start actions like reducing the access of cars to city centers, to mitigate the impact on the public health.

Among the different types of gas sensors, chemoresistive sensors have been attracting great interest due to their high sensitivity, low cost, reproducibility and simplicity [3]. Other techniques, like gas chromatography, electrochemical, and optical sensors, present some drawbacks, because they are more expensive, require trained personnel and still present some problems to miniaturize and export the measurement systems out of the laboratory [4,5]. However, chemoresistive sensors can be easily adapted to in-field measurements with low power consumption, especially when these sensors work at room temperature [6], reducing the complexity of the sensor driving circuitry. Nevertheless, selectivity is still an unsolved issue, especially regarding metal oxides [7]. Some approaches used arrays of sensors to mitigate this problem, but usually this alternative is more expensive and increases the complexity of sensor design and data treatment [8].

Many authors have developed chemoresistive gas sensors employing graphene semiconductors, however the use of pristine graphene for developing gas sensors still presents many intrinsic problems, including low gas sensitivity and complicated processability into devices [9]. On the one hand, the pre-processing and exfoliation of pristine graphene is complicated, for that reason oxidizing treatments (that ease the suspension of the nanomaterial in aqueous solutions)are employed [10], which are sometimes followed by reducing treatments [11]. These have opened the possibility of developing graphene oxide or reduced graphene oxide-based sensors, respectively. On the other hand, the interaction of gas molecules with pristine graphene is characterized by low adsorption energies and small charge transfer, especially when pristine graphene is operated at room temperature [12]. In consequence, different strategies have been developed, such as decorating graphene with metal or metal oxide nanoparticles [13,14] creating hybrid nanomaterials employing graphene and polymers [15] or via grafting organic molecules [16]. These modifications of graphene have been shown as good options to improve some sensing properties of graphene such as gas sensitivity, selectivity or response and recovery times.

Unlike metal oxides based sensors, which usually require high working temperatures to activate the oxidation-reduction processes and achieve the required electron mobility involved in gas sensing [17], graphene has been proved as a great option to develop room temperature based sensors [18], thanks to its extraordinary high carrier density and mobility even in the absence of heating [19]. This characteristic makes graphene an ideal candidate to be loaded with perovskite nanocrystals, another promising nanomaterial with exceptional properties such as large absorption coefficient, long carrier lifetime and high carrier mobility [20,21]. Perovskites require low operating temperatures to avoid degradation [22]. Some works reported the use of perovskite films as gas sensors [23,24], showing interesting results in the detection of NH_3_ [25], acetone [26], oxygen [27], and NO_2_ [28]. However, there are still some issues related to their fast degradation, high humidity, cross-sensitivity, and moderate gas sensitivity obtained [25].

Recently, graphene was successfully implemented in perovskite solar cells, improving these devices due to the high conductivity, enhanced charge carrier mobility and material stability conferred by graphene in electronic devices [29]. The stability is the main drawback of organolead perovskites due to their high sensitivity to atmospheric conditions. For instance, CH_3_NH_3_PbI_3_ (MAPbI_3_) usually shows a fast degradation, in most cases attributed to their hygroscopic properties [30], which result in the formation of their hydrates. This degradation is partially reversible upon removal of humidity [31]. However, the hydrophobic properties of graphene-based devices should mitigate one of the main problems using perovskites, which is the degradation in humid atmospheres [32]. In this paper, we evaluate, for the first time, the improvements in gas sensing performance achieved by decorating graphene with perovskites, showing high potential of the resulting hybrid nanomaterial for enhancing the sensitivity and selectivity to pollutant gases, and for blocking the perovskite degradation under the presence of ambient moisture. Here we employ a bromide perovskite (MAPbBr_3_), which has been proved to be more resilient to the presence of ambient moisture [33]. Then, this work tries to open a new approach to employ perovskite gas sensorss in real applications by enhancing the MAPbBr_3_ stability and increasing the sensor time-life, via limiting perovskite degradation. Moreover, the nanocomposite was characterized by several techniques, such as X-Ray photoelectron spectroscopy (XPS), high-resolution transmission electron microscopy (HRTEM), Field Emission Scanning Electron Microscope (FESEM), and Raman spectroscopy. In consequence, this paper shows the results with graphene decorated by MAPbBr_3_ NCs, registering higher sensitivities and detecting lower concentrations than other gas sensors based on perovskite thin films or similar approaches (see Table 1).

Threshold limit values (TLV) to 1-h exposure to NO_2_ are established at 200 ppb and 100 ppb, by the European Union [37] and the Environmental Protection Agency of the US [38], respectively. Furthermore, the average yearly limit mean for NO_2_ exposure is set at 40 ppb in the EU and at 53 ppb in the US. Maximum exposure values for NH_3_ are defined as the ST (short-term) and TWA (Time-Weighted Average) by the European Agency for Safety and Health at Work (EU-OSHA) [39] and by the National Institute for Occupational Safety and Health (NIOSH) in the US [40]. These data are summarized in Table 2. TWA means the average exposure of 8 h/day and ST is referred to short exposures of 15 min. Both institutions define similar ST and TWA values for NH_3_ limits. Thus, the development of sensor networks able to detect these trace concentration levels in a wide range of locations would be highly advisable. In consequence, sensors should offer reversible interaction with gas molecules for allowing the continuous monitoring of these species at an affordable cost.

## 2. Materials and Methods

### 2.1. Perovskite Synthesis and Graphene Preparation

To synthesize lead halide perovskite (MAPbBr_3_) nanocrystals, the method proposed by Schmidt, et al. [41] was followed, in which a 2 mL solution of 1-octadecene (ODE) was prepared and 85 mg of oleic acid (OLA) were added. Then, the solution was stirred and heated up to 80 °C in a hotplate and 33.5 mg of octylammonium bromide (OABr) were added. Continuously, both, a solution of methyl-ammonium bromide (MABr) in dimethylformamide (DMF) and another with lead (II) bromide (PbBr_2_) were mixed in the same solvent. Subsequently, these two solutions were added to the main solution and was cooled to 60 °C. Finally, 5 mL of acetone was added to the final solution to create precipitates of perovskite nanocrystals, which were separated from the unreactive solution by using a centrifugation technique at 6000 rpm during 10 min. Afterwards, the nanocrystals obtained were suspended in toluene.

A graphene solution was prepared using 1 mg of graphene nanoplatelets from Strem Chemicals, Inc. (USA) dispersed in 1 mL of toluene. Then, graphene nanoplatelets were sonicated employing a Sonic Tip (FisherbrandTM Model 705) at 40% of 700W for 1 h and 30 min using a 1 s on −2 s off pulsed sonication.

### 2.2. Material Characterization

The exfoliated graphene and the lead halide perovskite were characterized by several techniques such as XPS, HRTEM, FESEM and Raman spectroscopy.

The chemical composition of the nanomaterials obtained was studied via XPS, using a SPECS spectrometer (Berlin, Germany) equipped with a Phoibos 150 MCD detector, using a non-monochromatic X-ray source (Al) operating at 200 W. The intensity ratios of the different components were calculated from the area peak after a correction by the transition function of the spectrometer and a non-linear Shirley-type background subtraction. Additionally, graphene crystallinity was evaluated employing a Raman spectrometer from Renishaw, plc. (Wotton-under-Edge, UK), coupled to a confocal Leica DM2500 microscope. The laser used had a wavelength of 514 nm.

The morphology was studied employing a JEOL JEM 2100F (Tokio, Japan) HRTEM, at an operating voltage of 200 kV. Nanomaterials were ultrasonically dispersed in toluene and a drop of dispersion was deposited onto a carbon-coated copper grid, drying it at room temperature. Graphene porosity and perovskites NCs distribution were analyzed by Carl Zeiss AG - ULTRA 55 (Oberkochen, Germany) Field Emission Scanning Electron Microscope.

### 2.3. Sensor Fabrication

The materials obtained were deposited onto 1.5 cm^2^ quartz substrates previously cleaned in an ultrasonic bath, using an anionic detergent (Alconox) and miliQ water. Afterwards, a 1M solution of hydrochloric acid was employed in order to increase the surface hydrophilicity. Finally, the substrates were cleaned and dried several times with ethanol. Then, a film of graphene nanoplatelets was deposited onto quartz substrates by drop casting method, and subsequently, perovskite nanocrystals (5% wt solution) were deposited by spin coating at 1000 rpm for 120 s. 

Once the hybrid material film was deposited onto quartz substrate, its backside was glued with a thermally conducting epoxy from Heraeus, Inc. (Hanau, Germany) to an alumina hotplate that comprised a screen-printed platinum heater. Then, the samples were placed on a 20 × 30 mm printed circuit board (PCB) to be connected to an airtight test chamber. Finally, two-wire contacts were made on the sensor surface employing a conductive silver paste and platinum wires (a picture of a typical sensor is shown in the supporting information, see Figure S1).

### 2.4. Gas sensing Measurements

The resistance changes under different gases and experimental conditions were monitored using an Agilent HP 34972A multimeter connected to the gas sensing chamber. Additionally, calibrated gas cylinders were employed to apply synthetic air (Air Premier Purity: 99,995%) and different dilutions of gases tested to achieve the target concentration. In order to reproduce real atmospheric conditions, NO_2_ and NH_3_ gases were balanced in synthetic air as well. The total flow was adjusted to 100 mL/min using a set of Bronkhorst High-Tech B.V. (Ruurlo, The Netherlands) mass-flows controllers. The sensors were stabilized under synthetic dry air for 60 min before the application of the desired gas concentration during 30 min of exposure. Besides, a controller evaporator mixer (CEM) from Bronkhorst High-Tech B.V. (Ruurlo, The Netherlands) was used to humidify the gas mixture and thus, analyze the effect of ambient moisture on the sensing properties (see Figure S2). The relative humidity level was monitored by using a SHT51 humidity sensor from Sensirion AG (Stäfa, Switzerland) placed in a 35 cm^3^ airtight chamber. The ambient temperature inside the test chamber throughout the measurement process was 23 °C ± 1 °C.

## 3. Results

### 3.1. Material Characterization

**Figure 1:**
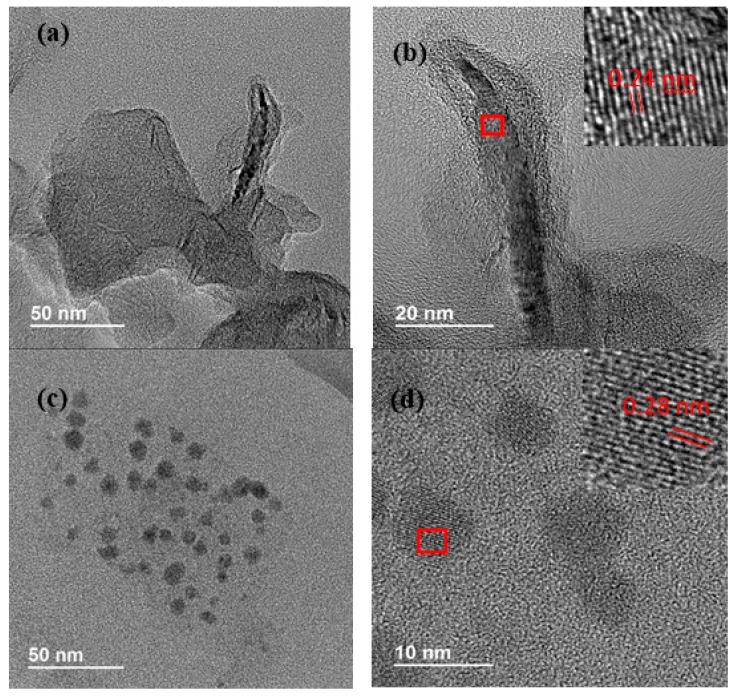
(**a**) HR-TEM image showing an example of the graphene layers size used. (**b**) HR-TEM image showing the graphene crystallinity. (**c**) Lead halide perovskites (MAPbBr3_3_) nanocrystals. (**d**) HR-TEM image showing the MAPbBr_3_ NCs crystallinity.

**Figure 2:**
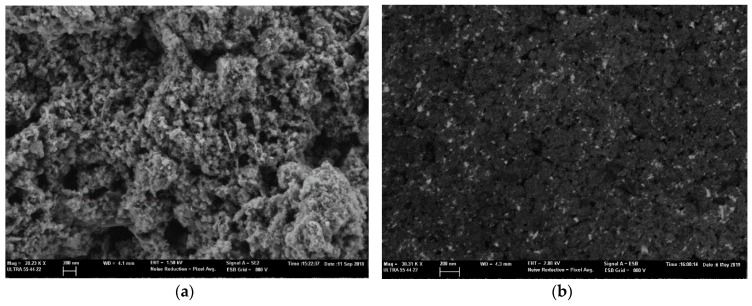
(**a**) FESEM image showing the sensor surface composed only by graphene. (**b**) FESEM image recorded with Back-Scattered Electron (BSE) detector, showing the graphene (black background) decorated with perovskite nanocrystals (bright spots).

A high-resolution transmission electron microscopy (HR-TEM) analysis was conducted to characterize graphene and perovskite nanocrystals independently. Figure 1a shows graphene layers with diameters near hundreds of nanometers. The interplanar distance obtained was 2.4 Angstrom (Figure 1b), showing a suitable graphene crystallinity. The perovskite synthesis method produces small nanocrystals, these lead halide perovskite nanocrystals to appear as dark spots (Figure 1c) with an average size of 7.1 ± 2.2 nm (see Figure S3 in the Supplementary Materials). Figure 1d shows the high crystallinity of the perovskites synthesized (Figure 2b), with a calculated interplanar distance of 2.8 Angstrom.

Additionally, FESEM images were obtained for both, bare and graphene decorated with MAPbBr_3_ NCs. Figure 2a shows the graphene once it was deposited onto the quartz substrates to be employed as a gas sensor, presenting a porous surface, which is interesting for gas sensing. Besides, in Figure 2b, the graphene decorated with perovskites nanocrystals can be observed, corresponding to the bright spots. To obtain this image, a Back-Scattered Electron detector (BSE) was used, revealing a quite homogeneous distribution of the nanocrystals on the surface of graphene.

**Figure 3:**
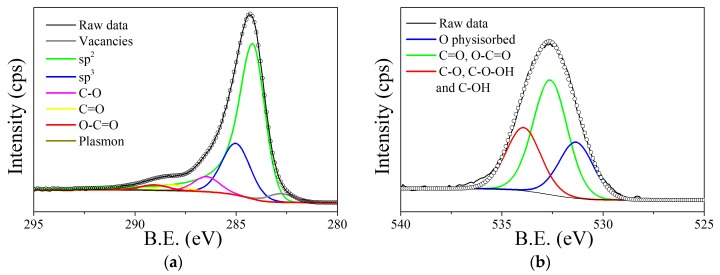
Deconvolution of the C 1s (**a**) and O 1s (**b**) core level peak for graphene.

According to the XPS elemental quantification, the graphene used presents a 7.8% content of oxygen and 92.2% of carbon. The results of the XPS fitting analysis are represented in Figure 3. The C1s spectrum, centered at 284.35 eV, is deconvoluted in seven peaks (Figure 3a). The component at 284.2 eV is characteristic of photoelectrons emitted from carbon atoms, associated with sp^2^ carbon systems in the “graphite-like” [42]. The peak reproduced at 290.5 eV is also characteristic of sp^2^ carbon systems, but in this case due to the π-π* interactions and π plasmon, which is derived from the energy loss due to the excitation of π electrons [43]. Components associated with carbon in amorphous or sp^3^ configuration appear at 285.0 eV [44], and defects associated with carbon vacancies appear at 282.9eV [45]. Meanwhile, associated to carbon-oxygen bonds, three peaks can be observed at 286.5 eV, 288.1 eV and 289.0 eV, which correspond to C-O, C=O and carboxylic groups, respectively [46]. Peak quantification is summarized in Tables S1 and S2 (see the supporting information).

The O 1s spectrum is reproduced employing three components centered at different binding energies, which are 531.3 eV, 532.6 eV and 533.9 eV (Figure 3b). The first one at 531.3 eV is attributed to oxygen physically absorbed, meanwhile the two peaks centered at 532.3 eV is associated to C=O, O-C=O and isolated OH, the peaks at 533.9 eV should correspond to C-O, C-O-OH and C-OH groups [47]. These oxygenated species will help to anchor and stabilize the perovskites in the graphene layers, similarly to the typical decoration of carbon nanomaterials with nanoparticles of different nature. Additional Raman Spectroscopy analysis was conducted to confirm the crystallinity reported before and after loading graphene with the perovskite (see Figure S4 Supplementary Materials).

### 3.2. Gas Sensing Results

**Figure 4:**
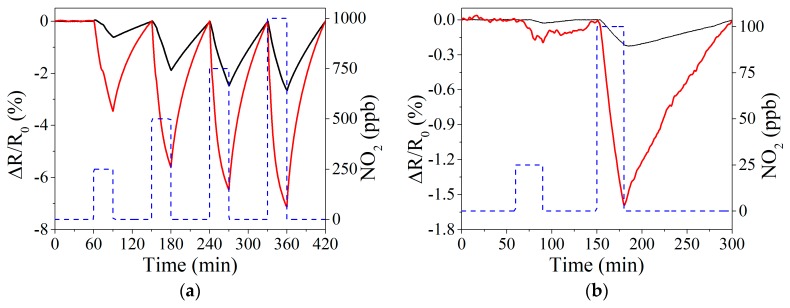
Example of resistance response when detecting NO_2_ at room temperature in the range of 250–1000 ppb (**a**) and 25–100 ppb (**b**). In both figures, black and red line corresponds to bare graphene and perovskite doped graphene, respectively. The concentration of NO_2_ applied is shown in right-Y, represented by a blue dashed line.

Two different gases, nitrogen dioxide and ammonia, with high interest to be monitored due to the danger to human health derived from their exposure at certain levels were measured. Several dilutions were performed in order to apply different concentrations, especially under the threshold limits. Due to the different baseline levels of the two films studied, normalized responses are show for better comparison in Figure 4. Also, it can be expected that a slight baseline drift appears, given the difficulty to completely clean the surface from adsorbed gas molecules due to the room temperature working conditions. However, a temperature treatment was performed on a weekly basis, to counteract drift, in which the sensors are heated up to 80 °C during 1 h to desorb the gas molecules at the surface and regain the original baseline.

Nitrogen dioxide detection was performed by applying repeated exposure and recovery cycles to increasing concentrations of the analyte considered. Figure 4a shows the response to 250, 500, 750, and 1000 ppb, in which graphene loaded with perovskite nanocrystals obtained higher responses compared to bare graphene. Due to the high responses obtained in this range of concentrations, especially for perovskite-loaded graphene, the concentrations measured were decreased in order to analyze the sensitivity under a few ppb of NO_2_. Figure 4b shows the responses obtained at 25 and 100 ppb, under room temperature conditions.

**Figure 5:**
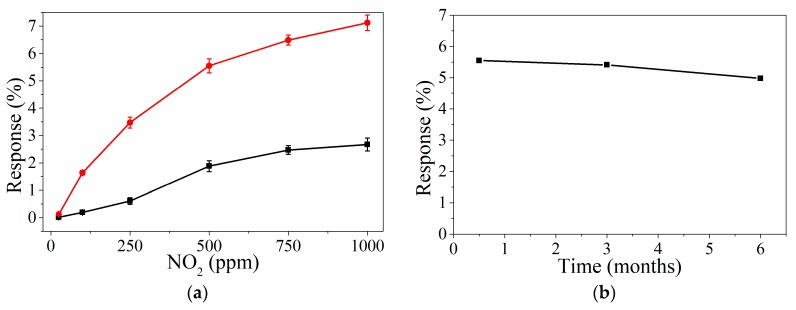
Calibration curves obtained for bare graphene (black) and perovskite doped graphene (red) detecting NO_2_ at ppb range (**a**). Stability study of the sensor based on graphene loaded with perovskite nanocrystals, 500 ppb of NO_2_ are measured over a 6-month period (**b**).

Figure 4 shows that response dynamics are slow and the saturation of the response is not achieved during the 30-min gas exposure. This drawback is often experienced with carbon nanomaterial chemoresistors, especially for room-temperature operated sensors. Slow response dynamics can be ameliorated by reducing the dead volume of the test chamber or by increasing the gas flow. Sensor response was computed as R(%) = 100*(R−R_0_)/R_0_, Where R_0_ is the resistance value immediately before gas exposure (i.e., under dry air), and R is the resistance immediately before the start of a new cleaning step (in other words, the last resistance value acquired while under exposure to NO_2_ or NH_3_). Figure 5 shows the calibration curves obtained for the whole range of NO_2_ concentrations measured. Graphene doped with perovskite nanocrystals presents a higher response (up to 3-fold) than bare graphene, even under a hundred ppb of NO_2_ exposure. Additionally, bare graphene presents a slight saturation above 500 ppb, decreasing the slope obtained and, in consequence, reducing sensitivity. 

A stability study of graphene loaded with perovskite nanocrystals was conducted (Figure 5b), revealing a slight decrease in the response towards 500 ppb of NO_2_ after six months of sensor operation. This result confirms the high stability of MAPbBr_3_ under ambient moisture and reactive gases, probably due to the nanocrystal structure of the perovskite. Zhu et al. [28] report the high stability of perovskites in nanocrystal structure over other alternatives. Besides, in our work, the perovskites are probably protected by the hydrophobic properties of graphene.

**Figure 6:**
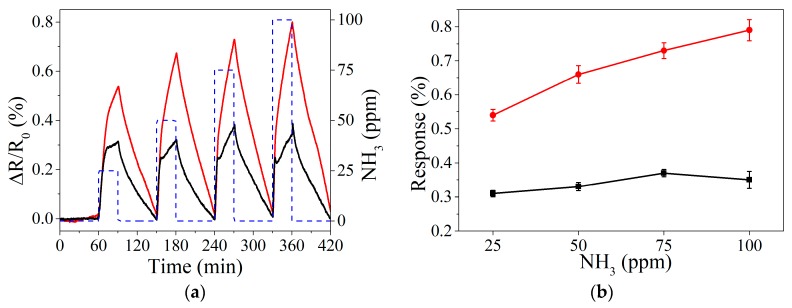
Example of resistance response when detecting NH_3_ at room temperature in ppm range (**a**). Calibration curves obtained for bare graphene and perovskite doped graphene, black and red lines, respectively (**b**).

The process and experimental conditions followed for NO_2_ detection, were applied as well for NH_3_ detection at ppm range (Figure 6a). Similarly, to the detection of nitrogen dioxide, ammonia measurements reveal a higher sensitivity for graphene decorated with perovskite nanocrystals (Figure 6b). Besides, meanwhile bare graphene presents a saturated response, decorated graphene shows a high potential to enhance the response under longer exposures to the analytes. However, an analysis of the steady-state reveals that several hours were not enough to get the saturation of the perovskite decorated graphene (Figure S5) at room temperature.

**Figure 7:**
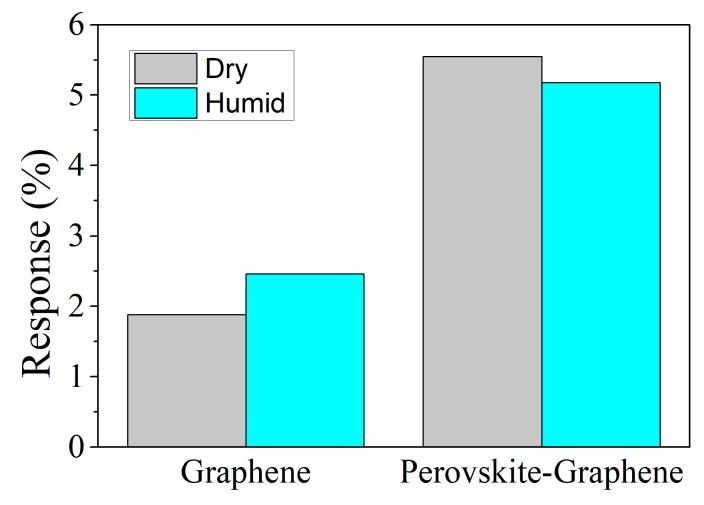
Comparison of the response for 500 ppb of NO_2_ under dry and humid (50% of relative humidity) conditions for bare and perovskite-decorated graphene.

Additionally, the sensitivity under dry and humid conditions was evaluated to both, bare and perovskite loaded graphene. Figure 7 shows an increase in the response for graphene to 500 ppb of NO_2_ (about 30%) when moisture is present. However, graphene decorated with perovskite nanocrystals shows a very stable response with only a slight decrease (about 5%) when under humid conditions.

## 4. Discussion

Graphene with oxygen-containing functional groups such as carbonyl, hydroxyl, epoxy, and ether groups exhibited enhanced hole transport characteristics due to its suitable work function [48,49]. In addition, it is well known that molecules adsorbed on the surface of graphene can change the local carrier concentration [50]. In this regard, exposure to an electron-withdrawing gas, like NO_2_ leads to a lower sensor resistance. Conversely, an electron-donating gas, such as NH_3_, decreases the hole concentration resulting in a higher sensor resistance. In films decorated with perovskite nanocrystals, the p-type behavior of the sensor is defined as well by the graphene nanolayers (these are the dominant carrier transport material), meanwhile the MAPbBr_3_ NCs are ambipolar charge transporters [23]. In other words, perovskites can act as a p- or n-type semiconductor depending on the interaction between the sensor surface and the nature of the gas (electron-donating or electron-withdrawing).

Bare graphene samples offer a response to both, NO_2_ and NH_3_, due to the interaction of these gases with the oxygenated defects and functional groups located on the graphene nanosheets. However, all the measurements were done at room temperature, obtaining limited sensitivity derived from the small transfer of electronic charge between graphene and adsorbed gas molecules. Nevertheless, from the gas sensing tests, it was derived that the presence of MAPbBr_3_ NCs decorating the graphene nanolayers was advantageous for detecting NO_2_ and NH_3_ at room temperature. In addition, surface trap sites at the perovskite have been demonstrated to act as active sites for the gas sensing process. It is reported that a net positive charge is formed at the surface of the perovskite due to the loss of bromine and undercoordination of the Pb atom, favouring the perovskite to be sensitive to the environmental gases [23]. Regarding the baseline level in dry air, bare graphene shows a lower resistance baseline (3-fold) than the decorated one with MAPbBr_3_ NCs. Upon the formation of the hybrid perovskite the overall resistance of hybrid films is higher than that of bare graphene due to the intrinsic electrical properties of the perovskite nanocrystals.

**Figure 8:**
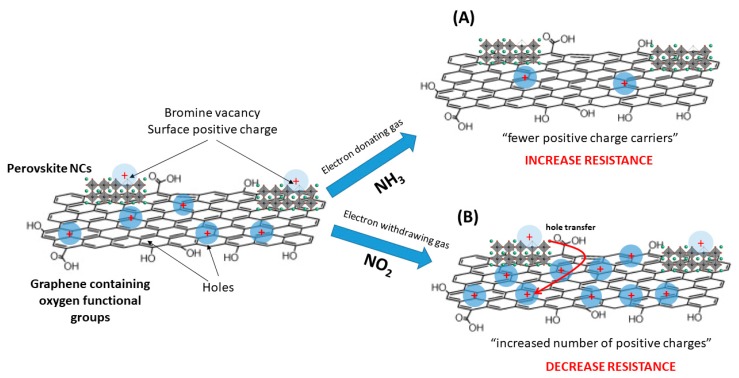
Schematic illustration of perovskite decorated graphene sensor showing the mechanism proposed after interaction with different gases. Two adsorption processes are proposed, one at the graphene surface and another at the perovskite NCs. (**A**) During the exposure to an electron-donating gas, an excess of positive charges is neutralized at the defective perovskite surface and the local hole concentration of the p-type graphene is decreased, which results in an increase in film resistance. (**B**) During the exposure to an electron-withdrawing gas, positive charges (holes) in the NCs are formed, which are transferred to the graphene layers from the NCs, decreasing the overall resistance of the hybrid film.

This enhancement in the response when graphene is decorated with MAPbBr_3_ NCs is associated to the creation of electron-hole pairs by the perovskites, once these are exposed to the gases (Figure 8). On the one hand, nitrogen dioxide is getting adsorbed on the graphene due to the interaction between the gas and the oxygen defects and functional groups of graphene. As a consequence, the hole concentration is increasing, resulting in a decrease in the resistance. Additionally, the perovskite NCs have a crucial role in the improvement of the sensitivity towards NO_2_, because the electron-hole pairs are separated, creating an interface between the graphene and the MAPbBr_3_. As a result, the excess of positive charges (holes) generated at perovskite NCs are transferred from the perovskite to the graphene sheet [51]. Therefore, the graphene film will suffer a large increase in the number of holes (and associated decrease in resistivity) when exposed to nitrogen dioxide, thanks to the presence of perovskite NCs. Inversely, when perovskite NCs initiate the creation of electron-hole pairs by the interaction with an electron-donating gas, in our case NH_3_, an excess of negative charges (electrons) are generated. As a consequence, perovskite NCs transfer electrons to graphene (and this is associated to an increase in resistivity), enhancing the response of the film to NH_3_ due to the higher concentration of electrons. 

One of the main drawbacks of the sensors based on perovskites films is the fast degradation derived from the exposure to ambient moisture. For that reason, a lead halide perovskite with bromide was chosen, due to the well-known stability over other perovskites based in halides like iodine [33]. Additionally, this paper reports perovskites in nanocrystal form, in front of the most usual approximation, based on films. With NCs, more stable perovskites can be obtained [28], enhancing the lifetime and durability of the sensor. Besides, the high hydrophobicity of the graphene can protect the MAPbBr_3_ NCs and slow down their degradation, due to the limited interaction with ambient moisture [30]. 

Considering the effect of ambient moisture in the sensing mechanism, higher sensitivity under humid conditions can be observed for bare graphene. This is in agreement with other works that report the use of graphene as humidity sensors [52]. Normally, water molecules act as electron-withdrawing, interacting with the oxygen functional groups grafted in the graphene. Besides, the detection of NO_2_ in humid conditions can be favored by a water-mediated adsorption, as reported in chemoresistive sensors [53].

Nevertheless, the detection of NO_2_ in the presence of ambient moisture for MAPbBr_3_ decorated graphene shows interesting characteristics. Figure 7 shows that a very similar response to 500 ppb of NO_2_ is achieved for hybrid sensors under dry and humid conditions. The response is only slightly lower in the presence of ambient moisture. Probably water molecules partly passivate the charge transport and the creation of electron-hole pairs of perovskite nanocrystals [54], slightly decreasing the improvement in the response registered by the presence of MAPbBr_3_ under dry conditions. The hydrophilic properties of perovskite decorated graphene were further studied by contact angle measurements (see Figure S6 in the Supplementary Materials).

The gas-sensitive layer developed shows its potential for detecting concentrations of nitrogen dioxide and ammonia at trace levels, even below their threshold limit values. These results are a promising beginning for the use of perovskite nanocrystal decorated graphene in gas sensing applications. However, selectivity should be further studied before their use under real conditions. One reasonable approach could be the development of multi-sensor arrays employing decorated graphene with different types of perovskite nanocrystals.

## 5. Conclusions 

This work reports the first use of graphene loaded with perovskite nanocrystals as a chemoresistive sensor. This nanocomposite material has been shown to be a good option to obtain gas sensors with high stability over time, avoiding the main problem associated with perovskites, which is their degradation in contact with ambient moisture. Decorated graphene nanolayers show enhanced nitrogen dioxide and ammonia sensitivity in comparison to their bare graphene counterparts and much reduced cross-sensitivity to the ambient moisture. Furthermore, a reversible response to NO_2_ and NH_3_ was achieved at room temperature, enabling the integration of these hybrid films in low-power gas sensing devices. In summary, perovskites can constitute an alternative to metals, metal oxides, polymers or other molecules commonly used in the modification of the surface of carbon nanomaterials such as graphene in view of tuning sensitivity and selectivity. 

## Figures and Tables

**Table 1:** Comparison of the sensitivity for different chemoresistive sensors that employ perovskites. NT = not tested. NO_2_ and NH_3_ sensitivities expressed as 10^−3^% ppb^−1^ and 10^−3^% ppm^−1^, respectively. Relative humidity (R.H.) effect expressed as the variation of the sensitivity. Degradation information about the stability of the perovskite during a few days or weeks. EDPIC = ethylenediamine lead iodide chloride.

	NO_2_	NH_3_	R.H. Effect	Degradation	Ref
**Graphene- MAPbBr_3_ NCs**	14.5	3.8	↓ 5%	No	This work
**MAPbI_3_ film- SCN^−^ ions**	0.53	3.9	↓ 11%	No	[26]
**MAPbBr_3_ film**	0.13	NT	NT	Yes	[28]
**NCNT- MAPbBr_3_**	0.88	NT	NT	NT	[34]
**MAPbI_3_ film**	0.62	11	↓ 16%	NT	[35]
EDPIC *	NT	4.3	NT	NT	[36]

**Table 2:** TLV for NO_2_ and NH_3_ exposure correlated with their averaging time. ST = short-time. TWA = Time-Weighted Average).

	Averaging Time	European Union	United States
**NO_2_**	1 h	200 ppb	100 ppb
	1 year	40 ppb	53 ppb
**NH_3_**	ST	50 ppm	35 ppm
	TWA	20 ppm	25 ppm

## Supplementary Materials

The following are available online at https://www.mdpi.com/1424-8220/19/20/4563/s1, Figure S1: sensor device images, Figure S2: schematic diagram of the gas sensing system set up, Figure S3: histogram based on the diameter of MAPbBr_3_ NCs, Figure S4: Raman spectra for graphene, Table S1: Graphene peak quantification of C 1s core level, Table S2: Graphene peak quantification of O 1s core level, Figure S5: Steady-state analysis, Figure S6: contact angle measurements.
