# Supplementary material for: Gas Sensing Properties of Perovskite Decorated Graphene at Room Temperature
Source: Sensors (Basel). 2019 Oct 20;19(20):4563. doi: 10.3390/s19204563 (PMC6832145; doi:10.3390/s19204563)
Supplement: Supplementary file 1 [file sensors-19-04563-s001.pdf]

SUPPLEMENTARY MATERIAL for

Gas Sensing Properties of Perovskite Decorated Graphene at Room Temperature

Juan Casanova-Cháfer [1], Rocío García-Aboal [2], Pedro Atienzar [2,*] and Eduard Llobet [1,*]

[1] MINOS-EMaS, Universitat Rovira i Virgili, 43007 Tarragona, Spain; juan.casanova@urv.cat
[2] Instituto de Tecnología Química, CSIC-UPV, Universitat Politècnica de València, 46022 Valencia, Spain; rogarab@itq.upv.es
* Correspondence: pedatcor@itq.upv.es (P.A.); eduard.llobet@urv.cat (E.L.);
Tel.: +34-963-877-805 (P.A.); +34-997-558-502 (E.L.)

Received: 27 September 2019; Accepted: 18 October 2019; Published: 20 October 2019

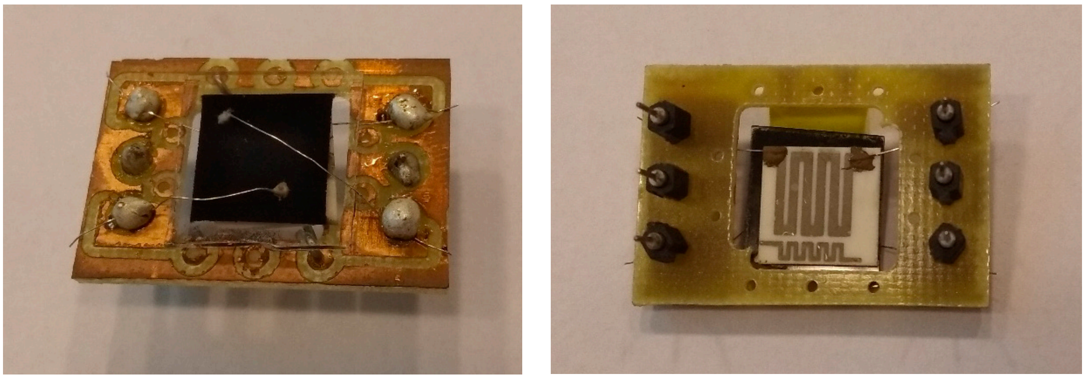

(a) (b)

Figure S1. Typical layout of a sensor device. Top view of the sensor showing the gas-sensitive coating and silver contacts (**a**) and backside of the device developed showing the alumina hotplate that comprises a platinum heating meander, which may be used to raise the operating temperature of the device above room temperature (**b**).

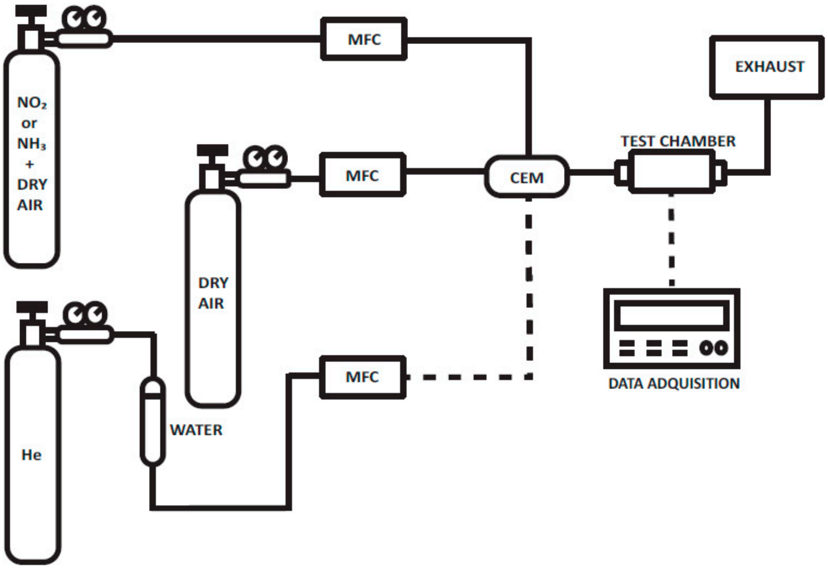

Figure S2. Schematic diagram of the gas sensing system set up. MFC: Mass Flow Controller; CEM: Control Evaporator Mixer.

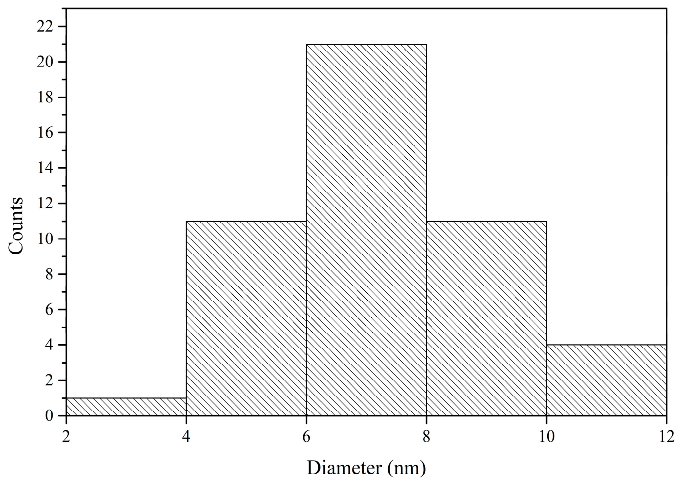

Figure 3. Histogram based on the diameter of perovskite nanocrystals. The most frequent size is situated between 6 nm and 8 nm, resulting an average diameter of 7.1 ± 2.2 nm.

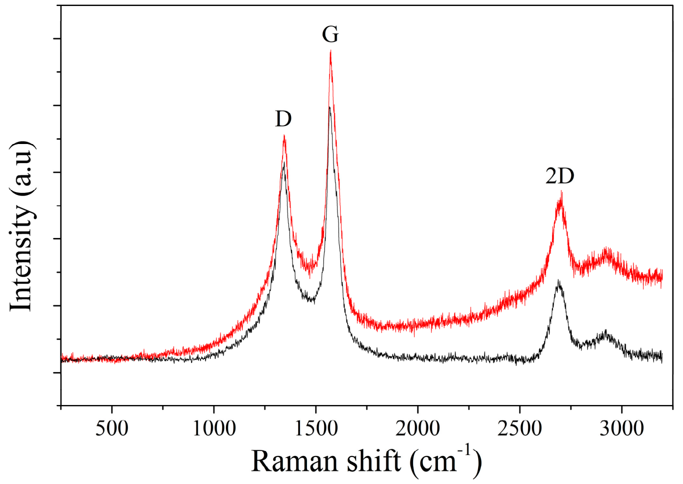

Figure 4. Raman spectrum obtained for graphene before (black line) and after (red line) being decorated with perovskite NCs. The graph shows the D and 2D peaks, related to the presence of defects in the sp^2 carbonaceous structure. Additionally, the G band corresponds to the in-plane vibrations of sp^2 carbon bonds. The intensity ratio between D/G bands is almost the same, decreasing from 0.78 to 0.74 after perovskite loading. Therefore, after the decoration of graphene, its crystallinity was preserved, as TEM images confirm. A drift is observed in the baseline after the decoration of graphene, probably because of the fluorescence derived from the presence of perovskite NCs.

Table S1. Peak quantification of C 1s core level peak for graphene.

Peak	Binding energy (eV)	Concentration (%)
Vacancies	282.9	3.80
Sp^2	284.2	59.76
Sp^3	285	24.88
C-O	286.5	5.84
C=O	288.1	3.30
O-C=O	289.0	2.06
Plasmon	290.5	0.36

Table 2. Peak quantification of O 1s core level peak for graphene.

Functional Group	Binding Energy (eV)	Concentration (%)
O-physically adsorbed	531.3	24.00
C=O, O-C=O and isolated OH	532.6	49.04
C-O, C-O-OH, C-OH	533.9	26.96

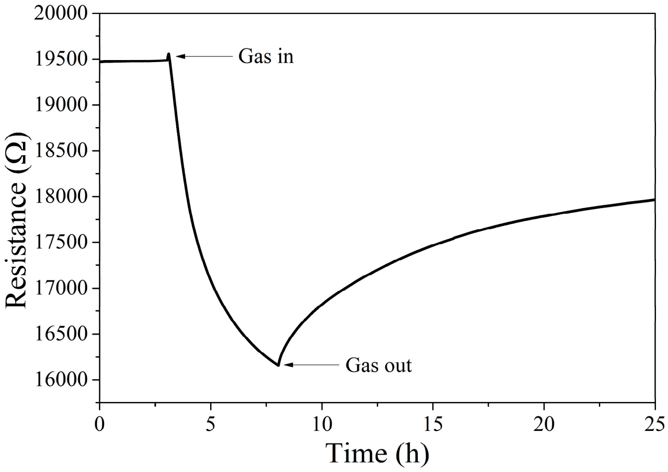

Figure S5. Steady-state analysis for a sensor operated at room temperature. Once the sensor surface is clean, 5 hours under 1 ppm of NO_2 were not enough to achieve the saturation of the response, probably due to the slow gas adsorption at room temperature. Additionally, more than 15 hours are needed to recover the baseline.

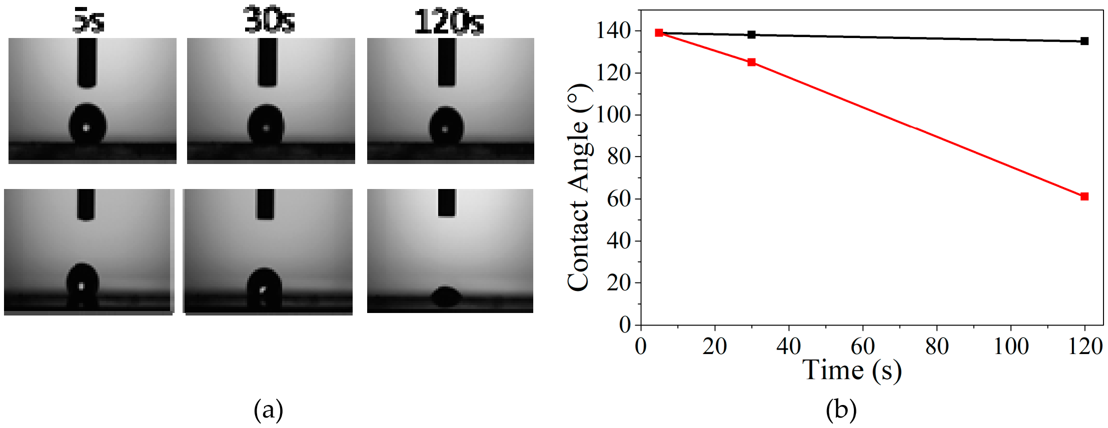

Figure S6. Contact angle measurement for non-decorated graphene (upper line) and MAPbBr3 decorated graphene (lower line) during 2 minutes (a). Summary of the evolution of the contact angle for decorated graphene (red line) and non-decorated (black line) (b).

Lead halide perovskites present strong hydrophilic properties, showing in most cases a fast degradation in contact with water molecules. Figure 8a shows the contact angle measurements when a drop of distilled water was deposited on the sensor surface. After 5 seconds, the contact angle is practically the same for bare and decorated graphene, probably because of the $MAPbBr_3$ nanocrystals are passivated, similarly to the effect observed in gas sensing measurements. However, meanwhile bare graphene keeps the contact angle high along time, Figure 8b shows a decrease in contact angle for $MAPbBr_3$ decorated graphene. Take note that in contrast to the conditions in which gas sensing measurements were performed under the presence of water vapor, in this experiment the hybrid material was exposed to significantly harsher conditions, since a drop

of water was directly deposited on the surface of the hybrid material. In consequence, liquid water could trigger the decomposition of perovskite NPs [1].

References

[1] Müller, C.; Glaser, T.; Plogmeyer, M.; Sendner, M.; Döring, S.; Bakulin, A.A.; Brzuska, C.; Scheer, R.; Pshenichnikov, M.S.; Kowalsky, W.; et al. Water Infiltration in Methylammonium Lead Iodide Perovskite: Fast and Inconspicuous. *Chem. Mater.* **2015**, *27*, 7835–7841. doi:10.1021/acs.chemmater.5b03883.
